# An Integrated Approach to Determining Rural Tourist Satisfaction Factors Using the IPA and Conjoint Analysis

**DOI:** 10.3390/ijerph16203848

**Published:** 2019-10-11

**Authors:** Yanchun Jin, Yoonseo Park

**Affiliations:** 1 School of Management, Eastern Liaoning University, Dandong 118001, China; 2 Department of Business Administration, Jeonbuk National University, Jeonju 54896, Korea

**Keywords:** importance-performance analysis, conjoint analysis, importance grid analysis method, sustainable rural tourism, tourist satisfaction

## Abstract

Rural tourists satisfaction has a pivotal role in the development of sustainable rural tourism. As a method of identifying critical satisfaction factors, an importance and performance analysis (IPA) technique has attracted growing interest from academics due to it being able to deliver the importance and performance of a product’s attributes from the standpoint of customers. However, IPA is based on the presumption that a linear and symmetrical relationship exists between the performance and overall satisfaction, which has been criticized by many researchers due to its deviation from the facts. On measurement of importance, researchers have not reached an agreement on whether direct or indirect approach should be applied. To measure satisfaction more effectively, this study presents a revised IPA method that integrates IPA, conjoint analysis and importance grid analysis. Based on mathematical psychology and psychometrics theory, the conjoint analysis method can be used to analyze multi-attributes of various products and derive relative importance of attributes in customer satisfaction research. The importance grid analysis method has been applied to categorize attributes by many researchers. It can be used to measure the nonlinear relationship between the performance of attributes and overall satisfaction. In this paper, an empirical study on rural tourists’ satisfaction was undertaken using this integrated method. The results show that the integrated approach is more responsive to attribute performance, thus allowing for improvement of a certain target attribute in the customer satisfaction enhancement process.

## 1. Introduction

Rural tourism has the potential as a development tool for rural areas [1]. As a dimension of developing sustainable tourism, satisfaction plays a critical role in the survival and future of tourism industry. Regarding the conservation of rural nature and culture, it is important to find the critical factors that have direct impacts on satisfaction for achieving sustainable rural tourism development. Among various studies on customer satisfaction, identification of customer satisfaction factors is regarded as essential because it can affect resources allocation on different service attributes for satisfaction improvement [2]. Product or service attributes are characteristics by which offerings are identified or differentiated, which usually include features, functions, benefits, and uses [3]. According to Lancaster [4], customers’ preferences are not on the product itself but on the characteristics or attributes of the product. Furthermore, product selection can be conceptualized as a process of comparing the main attributes of product or service. For this reason, investigating the critical attributes is a continuing concern within satisfaction research.

Using this approach, key attributes of products or service are generated first and then rated by customers according to their impacts on purchase decision. However, various methods have been proposed on the measurement of importance, and the agreement has not been acquired. Jaccard, Brinberg, and Ackerman [5] indicated that it is necessary to focus on the conceptual foundations of measurements of attributes importance through comparing six methods of importance measurements. Oliver [6] also suggested that customers should know “important for what” when they rate the importance of attributes. Furthermore, Oliver suggested that, instead of measuring importance alone, “incorporating the importance of performance into predictions of satisfaction is available”. This is in line with Martilla and James [7], who indicate that it is more effective to examine both importance and performance than focus on importance only. In addition, they introduced importance–performance analysis (IPA) that has been used widely in satisfaction research. Through investigation of an attribute’s importance before purchase and performance perception afterwards, the evaluation of satisfaction can be acquired.

Based on attributes approach, importance and performance analysis can derive practical suggestions through the measurement of attribute importance and performance. Due to its simplicity and effectiveness, the IPA technique has been widely used in many fields for analyzing service quality, destination image, market segmentation, destination competitiveness and so on [8,9,10,11,12]. However, according to Oh [13], the revision of a traditional IPA technique is necessary considering conceptual and practical issues. Conceptual issues involve the uncertainty of the criterion for measuring importance and performance; practical problems exist in the survey design and grid scale. Until now, various revised IPA techniques have been provided, but the definition of importance and relationship between importance and satisfaction are unclear. As an effective method in marketing research, continuous focus on the modification of IPA is necessary.

The aims of this study are to present a novel solution for the measurement of attribute importance in the IPA technique. As Oliver and Oh [6,13] have highlighted, we use satisfaction as the same criteria to measure importance and performance but with different methods. For the importance measurement, we use conjoint analysis to acquire relative importance of attributes. Different from this, performance is measured using the direct method. The second aim is to increase the utilities of IPA through adding the diagonal line and discrepancy analysis. In addition, a major criticism of IPA is its assumption that a symmetric relationship exists between attribute performance and satisfaction. In this study, the asymmetric relationship between attribute performance and satisfaction is analyzed.

The rest of this paper is organized as follows. The second section reviews previous literature about IPA and conjoint analysis research. The third section introduces a revised IPA approach to identifying the relative importance and performance of attributes using conjoint analysis and the importance grid method. The fourth section demonstrates the implementation of this proposed revised IPA framework in two rural tourism scenic spots in China. Conclusions are drawn in the last section.

## 2. Theoretical Background

### 2.1. Importance–Performance Analysis

Attributes are defined as the determinant decision criteria that can be used to evaluate products or services [14]. In Myers and Alpert’s research [15], attributes are identified as determinants that are closely related to customer’s preference or purchase decision. As a method based on the analysis of attributes, the IPA technique was introduced by John A. Martilla and John C. James in 1977 and firstly used in analyzing the service quality of automobile dealers. Through response to two questions of “how important is this feature” and “how well did the dealer perform” [7], the IPA grid was divided into four areas named “concentrate here”, “keep up the good work”, “low priority” and “possible overkill” can be acquired. The analysis result of attributes derived from IPA can help firms allocate funds more effectively on pivotal and critical attributes of products.

Due to its simplicity and effectiveness, IPA has enjoyed popularity. Its application has been broadened to a variety of areas, especially in research for identifying critical factors for product selection and evaluation of service quality and satisfaction [16]. As an extension, Sethna [17] has a related IPA technique for customer satisfaction and provided a hypothesis that the greater the discrepancy between importance and performance of a product on that attribute, the greater the customer’s dissatisfaction with the product. It has been proven to be an effective tool in research for identifying determinants affecting overall satisfaction [18]. Moreover, IPA instead of SERVQUAL has been suggested to measure service quality for limitations existed in the latter [9,19].

Although the ease of application and simplicity have led to the wide acceptance of the IPA research framework, researchers have disagreements on measurement of importance and asymmetrical effects between attributes’ performance and satisfaction [13,20,21,22]. Importance, also termed as “value importance” or “salience”, reflects customer’s preference or stress on different attributes. For measurement of importance, it has been heavily criticized for its ambiguous, multidimensional definitions in previous studies; in particular, both direct and indirect ratings of importance were used in previous research. The direct method is a simple method. However, it has apparent problems. To date, a variety of revised IPA research frameworks using indirect or integrated methods has been provided to avoid its disadvantages [23,24,25,26]. Multiple regression analysis, three factors theory, partial correlation analysis, and a back propagation neural network have been addressed for customer self-stated importance for avoiding problems such as incorrect interpretation of attributes’ importance and changing evaluation in different purchase stages [23,27,28,29]. The second disagreement on attribute research is the relationship between attribute performance and satisfaction. It has been reported that attribute performance and satisfaction have a positive association [30]. Asymmetrical effects of attribute-related performance on overall satisfaction have also been noticed [30,31]. The method of combining three-factor theory with the IPA technique for measuring asymmetrical effects has been used in many research works [23,27,32].

Normally, two steps are required to apply the IPA method. First, the importance of attributes is measured prior to actual purchase experience. The same set of attributes is then used to evaluate performance. However, if we use a random sampling method, it is impractical to make an investigation before and after the purchase on the same investigators. Therefore, some researchers have suggested that the investigation should be performed concerning the importance and performance at the same time after purchase. Neslin [33] has suggested that a statistical method should be employed rather than a self-stated method for predictive validity. However, this also has problems. The main criticism raised by other researchers is that, if these two factors (importance and performance) are evaluated at one time, close relations between importance and performance would appear. Moreover, all attributes to be evaluated are likely to be important. This would bring about “ceiling effects”. For the multi-dimensions of definition and measurement of the importance, Oliver [6] has also suggested the use of satisfaction as a measure criterion of importance and performance.

### 2.2. Conjoint Analysis in Satisfaction Research

Since being proposed by Green and Rao [34], the conjoint analysis method has been widely used in the field of marketing research. The objective of conjoint analysis is to quantify the choice process on products or services based on experimental design and various data collecting methods including ranking, rating or choice-based methods. Historically, the compositional approach was utilized on customers’ choice processes, with more attention given to attributes or characteristics of products in the 1970s and 1980s [35]. Different from this approach, conjoint analysis is a decompositional approach. It gets utilities of product attributes from alternatives or profiles that are made up of various attributes of products.

Because conjoint analysis method is more similar to a customer’s actual purchase decision or attribute evaluation, which is used in satisfaction research mainly on the measurement of attribute’s relative importance. Using the conjoint analysis method, attributes’ utilities and individual level utility can be derived, and the percentage of utility range is defined as relative importance. This can be seen in previous research [36]. Thus, the conjoint analysis technique is also applicable to drawing inference to the importance of attributes that can provide useful information to explain why people make different purchase decisions [37]. It is thus well accepted that this approach is an objective and realistic way to obtain the relative importance of attributes.

The conjoint analysis method can be used in IPA research framework based on these reasons: first, relative importance of attributes can be measured using the conjoint analysis method. It can be seen that a measurement of relative importance rather than direct ratings was suggested in previous research [13,38]. Through designing the profile in conjoint analysis, a customer’s real attitude on attributes can be reflected. It will be not influenced by perception of the attribute’s importance and performance. Second, orthogonal design is applied in the profile design of conjoint analysis, and the relationship among attributes appears to have zero correlation. Additionally, conjoint analysis “depends on less restrictive assumptions than multiple regression analysis” [21], and the limitations that exist in the reported revised IPA technique can be minimized.

## 3. Methodology of Conjoint Analysis Based on Importance–Performance Analysis

### 3.1. Acquiring of Implicitly Derived Importance and Performance

The traditional IPA technique employs customer self-stated importance and the performance approach using Likert five-point or seven-point rating scale. Thus, it is considered as a simple and well-understood method by both researchers and customers. However, various problems have arisen with the wide application of the IPA technique. For instance, a linear relationship between importance and performance was usually assumed, and every attribute was tended to be thought of as very important [20]. More recently, related studies have focused on implicitly derived importance and the performance approach. Matzler and Sauerwein [2] have provided the sensitivity of importance weights through comparing the implicitly and explicitly derived importance approaches. In line with previous research, this study analyzes the nonlinear relationship between attribute-level performance and overall satisfaction based on the implicitly derived importance.

### 3.2. The Revised IPA Procedure

Based on previous research, this study provides a new approach to helping managers derive a more precise and simply applied marketing strategy by using the IPA technique. First, as both self-stated importance and the implicitly derived method are questionable, this study employs the conjoint analysis method for deriving the relative importance of an attribute. Second, this study uses satisfaction ratings derived directly to acquire performance evaluation. The nonlinear relationship between attribute-level performance and overall satisfaction can then be delivered using importance grid analysis method. Finally, the attribute’s importance and performance will be plotted on the IPA grid.

#### 3.2.1. Step 1: Conjoint Analysis Design

Experimental design of a conjoint study includes several steps. Some critical ones are the identification of product attributes and levels, the determination of the analysis method such as ratings-based and choice-based methods, and the design of profiles. Until now, four types of methods have been used in conjoint analysis: (1) a traditional method that uses stated preferences, (2) choice-based conjoint analysis that uses stated choices, (3) adaptive conjoint analysis, and (4) self-explicated conjoint analysis [35]. However, full profiles or a smaller set of full files using stated preference are accepted widely due to a high applicative percentage in previous conjoint analysis studies.

For profile design, the orthogonal plan is supported by most researchers as it can avoid the multicollinearity among attributes effectively. In addition, it needs less profiles than the full-factorial design. However, the orthogonal plan can only measure the main effects of attributes while interactive effects will be ignored. Thus, fractional factorial design that can measure both main effects and interactive effects or higher-order effects is considered in this study. To satisfy all requirements, Box–Behnken design (BBD) is selected for data collection that will use 12 runs with three coded levels −1, 0 and 1 (Table 1).

#### 3.2.2. Step 2: Computation of Importance and Performance Values

One can compute the relative importance of the attribute by part-worth function that is specified as a piecewise linear function in dummy variables in conjoint analysis studies. According to Rao et al. [35], the component utility function for the *t*-th attribute can be written as:

(1) $$U_{t}\left(x_{jt}\right)=U_{t1}D_{t1}+U_{t2}D_{t2}+\ldots +U_{tr_{t-1}}D_{tr_{t-1}},$$

where $U_{tk}$ is the component of the part-worth function for the *k*-th level of $x_{t}$, $x_{jt}$ is the level for the *j*-th profile on the *t*-th attribute, $r_{t}$ is the number of discrete levels for the *t*-th attribute, and $D_{tk}$ is the dummy variable taking the value 1 or 0. This formula can be used to calculate the utility of attribute. Its relative importance can also be determined.

The evaluation of performance can be acquired through asking customers to rate the satisfaction of attributes. In most of the previous research, the five-point Likert scale of 1 (very dissatisfied), 2 (somewhat dissatisfied), 3 (neither dissatisfied nor satisfied), 4 (somewhat satisfied), and 5 (very satisfied) was used to rate performance of attributes.

#### 3.2.3. Step 3: Categorization of Attributes

Recent research supports the view that attributes have a nonlinear relationship with satisfaction. Depending on its impact on satisfaction, attributes can be categorized as basic attributes, excitement attributes, or performance attributes [39,40]. Basic attributes can respond to basic needs for the product or service. It will cause dissatisfaction if not fulfilled. However, it does not bring customer delight if exceeded. On the contrary, excitement attributes can increase customer satisfaction if delivered, although it does not cause dissatisfaction if not fulfilled [27]. Performance attributes will lead to satisfaction if the attribute performance is high. It will cause dissatisfaction if its performance is low.

Vavra [41] firstly proposed that the importance grid could be used to identify the three satisfaction factors (basic attributes, excitement attributes, and performance attributes). Importance grid is constructed depending on whether the importance of attribute is derived explicitly or implicitly. A customer’s self-stated importance is identified as explicit importance. It is the indicator of an attribute’s dissatisfaction-generating potential. Different from explicit importance, as an indicator of satisfaction-generating potential, implicit importance is obtained indirectly such as applying regressing attribute-level performance against overall satisfaction [2,42,43,44].

**Figure 1:**
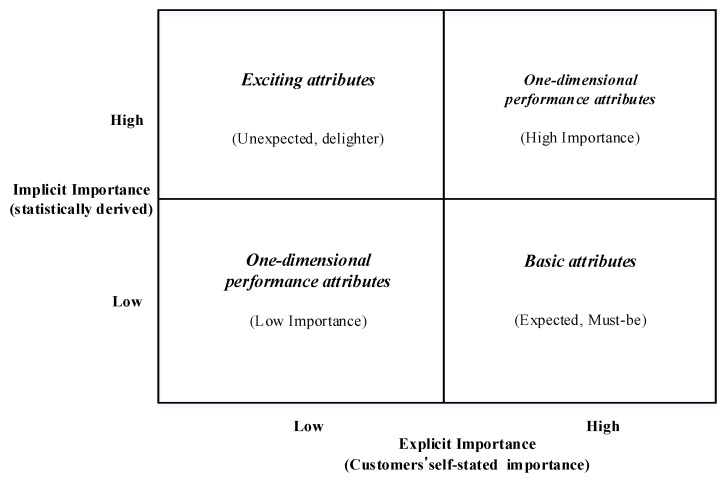
Importance grid for attributes (Vavra [41]).

The assumption of importance grid analysis is that explicit importance and implicit importance might differ in reflecting the importance-satisfaction relationship. In addition, it has been stated that a customer’s self-stated importance cannot measure the relative importance of attribute adequately [45]. Importance grid analysis combines attribute importance weights derived explicitly and implicitly in a two-dimensional grid. The attribute can be plotted according to differences in importance weights (Figure 1).

In terms of importance grid analysis, basic attributes are factors that have strong negative impact on overall satisfaction in low-level performance without having a significant positive impact when performance is high. It is a minimum requirement of product or service. Thus, it can be identified as high importance in directly derived evaluation of attributes, but as low importance in indirectly derived evaluation. Different from basic attributes, exciting attributes are identified as not much important in directly derived evaluation but as highly important in indirectly derived evaluation for its positive relationship with overall satisfaction on the high performance of attribute. For one-dimensional performance attributes, their corresponding changes can be shown between the performance of attribute and overall satisfaction. An attribute with high explicit and implicit importance can be considered high importance attributes. On the contrary, low importance attributes show little importance both in explicit and implicit ways.

#### 3.2.4. Step 4: Importance–Performance Grid Creation

Using importance and performance value derived from step 2, attributes can be plotted on the IPA matrix. For Importance–Performance (I–P) map partitioning, two types of quadrants approaches are mainly used. One is the “scale-centered quadrants approach”, suggested by Green and Rao [34]. The other is the “data-centered quadrants approach”, which uses empirical means obtained from the data as cross-points [46]. In addition, based on the traditional matrix, which divides the region into four parts to analyze characteristics of attributes, some researchers have added a diagonal line on the matrix for representing high priority for improvement more clearly. This method has been proven to be more effective than the traditional one [20].

In this study, the revised I–P matrix with data-centered quadrants approach and diagonal line is employed. Combined with analysis results of attributes’ category, improvement suggestions for attributes will be derived.

## 4. Implementation of Revised IPA

### 4.1. Study Area

Two villages in China named Dalishu and Qingshan were selected for this study. Both of these two villages are near the city and famous for rural tourism resources. However, tourist attraction in these two areas have their own characteristics. Zheng [47,48] evaluated the development of multifunctional agriculture in Dalishu village. Rural tourism was identified as an enhancement foundation of multifunctional agriculture and rural sustainable tourism through outdoor activities, fruit picking and dining experiences. Compared to Dalishu village, rural tourism in Qingshan village offers natural and Manchu cultural landscape as tourist attractions. Rather than experienced activities, landscape appreciation is more concentrated in Qingshan village.

The two rural scenic spots are named after the village directly. With both similarities and differences existing in these two scenic spots, it can examine the revised IPA framework more effectively on the management focus on the attributes and the relationship between attribute performance and satisfaction.

### 4.2. Questionnaire Design

According to the Organization of Economic Co-Operation and Development (OECD), rural tourism is defined as tourism taking place in the countryside: “rurality is the central and unique selling point in the rural tourism package” [49]. Previous studies on rural tourism expectation and motivation were mainly concentrated on relaxation, socialization, learning, family togetherness, novelty, and excitement [50], functional factors (i.e., reservation system, service quality) and technical factors (i.e., room size, price level) [49], and access evaluation, lodgings availability, and price evaluation [51]. Based on these previous studies considering the characteristics of rural tourism in China, this study initially selected six factors (transportation, price level, rural lodging, rural eating facilities, rurality experience activity and rural tourism service quality) as expectation and satisfaction factors. However, after consulting with tourism researchers and travel agency managers, we decided to delete three factors: transportation, rural lodging and rural eating facilities. The reason to delete transportation is that transportation evaluation includes many aspects, such as time, price, and comfortability that will make respondents feel that they are difficult to evaluate. The reason to delete the other two factors is because the location of the survey region, one of which is very close to the city, and the majority of tourists will not have the experience of rural eating or rural lodging experience. Therefore, the three factors determined in this study are rural tourism price level, rurality experience activity and rural tourism service.

Regarding levels of attributes, we selected satisfaction as the measurement of the importance of attributes based on Oliver’s suggestion [6] and Danaher’s research [38] as shown in Table 2.

This experiment design has three factors each with three levels, resulting in $3^{3}=27$ treatments which would be too many for respondents to evaluate. Different from previous research, this study applies Box–Behnken design that is evaluated as a very effective design method for researching the relationship among variables. For the case of three factors with three levels each, it needs 12 experiment runs. Therefore, we designed 12 questions according to the BBD method, and used five-point Likert scale (1 for “very dissatisfied” and 5 for “very satisfied”) to evaluate the satisfaction of the combination of three attributes with different levels.

Holdout cases are generated randomly for checking the internal validity of the model. They are judged by respondents but not used in the conjoint analysis. According to previous studies, we used four holdouts that were mixed into the 12 questions randomly. Consequently, each respondent was asked to rank 16 alternatives.

### 4.3. Data Collection and Respondents’ Profiles

The questionnaire was administrated online with a snowball sampling approach to residents in Dandong City who had already participated in rural tourism in Dalishu and Qingshan scenic spots. This approach is chosen here since tourists mainly come from regions near the city. This survey integrated persons from those urban areas known to generate the most rural tourists in Dandong city as well as tourist guides who were asked to invite tourists who had the experiences to these two scenic spots to participate in the survey. The award for this survey is the chance of a drawing in a lottery that is supported by an online questionnaire design company.

The survey was conducted from October to November 2018. A total of 155 questionnaires were received. Questionnaires from respondents who finished this survey in less than three minutes or selected the same options for all of questions were deleted. Finally, 115 valid and usable questionnaires were used for analysis. Demographic profiles of these respondents are summarized in Table 3.

### 4.4. Reliability and Validity Analysis

Although there have been disagreements on the reliability and validity evaluation of conjoint analysis, Pearson’s r and Kendall’s tau τ statistics based on holdout samples as simple and effective measurement methods are widely used [52]. They are reported as indicators of fit between the model and obtained data. Pearson’s r can be used to measure the degree of correlation between attribute levels within a factor. Kendall’s tau is a measurement of the correlation between the observed and the predicted preferences of rank order variables.

We analyzed the internal reliability of the two scenic spots, respectively. Results are shown in Table 4. Test results showed very high overall correlations with correlation coefficient r of 0.945 for Dalishu scenic spots and 0.950 for Qingshan scenic spots. Kendall’s tau τ was 0.870 for Dalishu scenic spots and 0.818 for Qingshan scenic spots in all conjoint models, indicating a good and efficient model fit. For the four holdouts cards, the Kendall’s tau τ statistics confirmed the model’s reliability both at 0.333 in the two scenic spots. It showed cross-validity about the model’s ability to predict ratings of hold-out profiles.

### 4.5. Implicitly Derived Importance and Performance of Tourist Satisfaction Attributes and Dimensions

#### 4.5.1. Conjoint Analysis of the Two Rural Tourism Destinations

**Figure 2:**
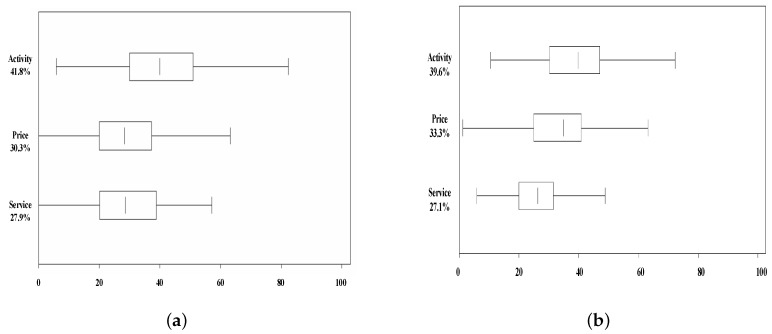
Relative importance of attributes ((**a**) Dalishu scenic spots; (**b**) Qingshan scenic spots).

**Figure 3:**
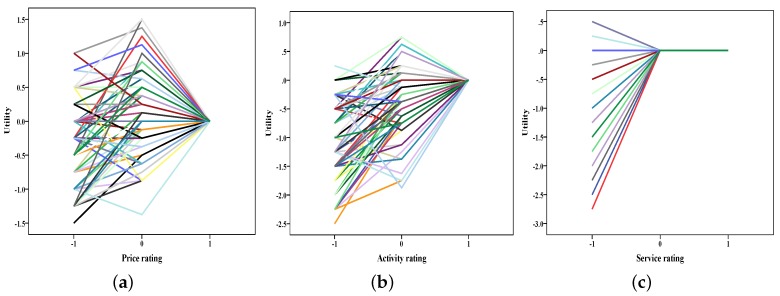
Utility plot of attributes in Dalishu scenic spots ((**a**) utility change with price rating; (**b**) utility change with activity rating; (**c**) utility change with service rating).

**Figure 4:**
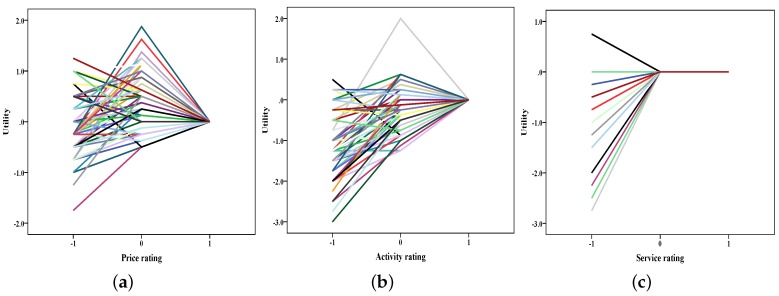
The utility plot of the attributes in Qingshan scenic spots ((**a**) utility change with price rating; (**b**) utility change with activity rating; (**c**) utility change with service rating).

Using SAS procedure Conjoint Analysis, we obtained relative importance values of the three attributes, respectively. Relative importance values of the three attributes [rural tourism product price level (price), rurality experience activity (activity), and rural tourism service (service)] were 0.303, 0.418, 0.279 in Dalishu and 0.271, 0.396, 0.333 in Qingshan scenic spots (Figure 2). Furthermore, we analyzed personal utility of attributes with different levels. Results are shown in Figure 3 and Figure 4.

These attributes were coded as −1, 0, and 1 to represent “worse than expected”, “about what was expected” and “better than expected”, respectively. According to customers’ ratings, utilities of attributes can be acquired. Changes of utilities with attributes’ ratings seem interesting as shown in the two figures. When ratings of attributes changed in “worse than expected”, “about what was expected” and “better than expected” three levels, utilities did not seem to increase continuously. Especially for service attributes, it brought the same utility regardless of the “about what was expected” level or the "better than expected” level. Similar results were also seen in previous research that addressed an asymmetry relationship between service and overall satisfaction.

#### 4.5.2. Creation of Importance Grid

Using the importance grid analysis method, the classification of attributes can be obtained. First, the importance of attribute derived directly can be acquired from questionnaires such as explicit importance and the relative importance obtained from conjoint analysis is used as implicit importance. Results are shown in Table 5.

**Figure 5:**
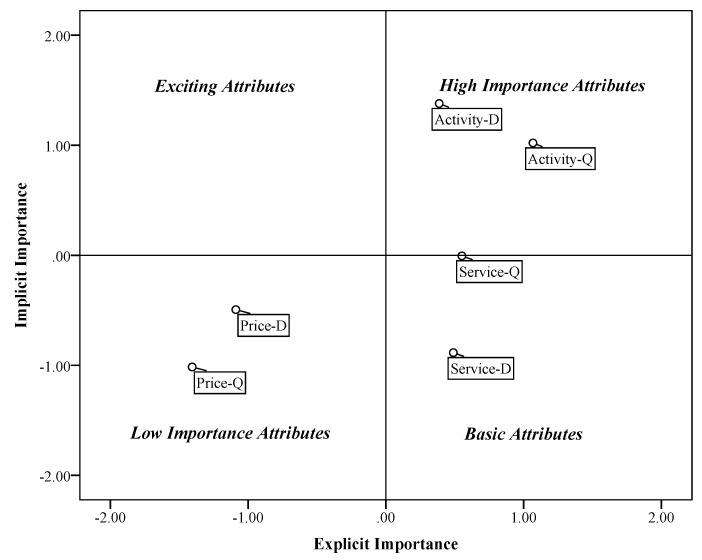
Importance grid for attributes (D: Dalishu scenic spots; Q: Qingshan scenic spots).

Grand means of explicit and implicit attribute importance are used as the axis of the plot. Attributes are then plotted in the grid (Figure 5). The “service” attribute that has high explicit and low implicit importance is categorized as the basic attribute of overall customer satisfaction. “Price”, which has low explicit and implicit importance, is considered a low important attribute. On the contrary, “activity”, which has high explicit importance and high importance, is categorized as a high importance attribute.

Since “activity” is classified as a high importance attribute, it suggests that improvement of efforts and special attention should be given to help enhance the satisfaction of tourism activity. Since “tourism service” is a basic factor, it suggests that business managers should pay more attention to keeping the existing level and minimizing the cost of tourism service.

### 4.6. Creation of Importance–Performance Analysis Grid

The relative importance of attributes obtained from the conjoint analysis and the performance value obtained directly will be plotted on the IPA grid. Table 6 shows results of importance value, performance value, and corresponding values for discrepancy for Dalishu and Qingshan scenic spots. Corresponding values for discrepancy are obtained by calculating the difference between performance and importance using their standardized value shown in the brackets. As shown in Table 6, "rurality experience activity” of Dalishu scenic spots and "rural tourism service” of Qingshan scenic spots had the biggest negative discrepancies. In contrast, the "rural tourism product price level” of the two scenic spots presented a clear positive discrepancy.

**Figure 6:**
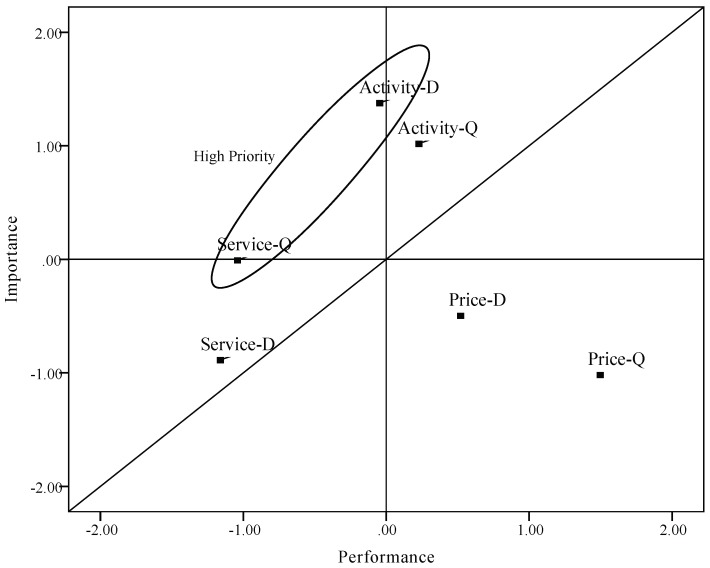
Importance-performance grid for attributes (D: Dalishu scenic spots; Q: Qingshan scenic spots).

We then standardized the value of importance and performance to avoid problems existing in “scale-centered quadrants”. Point (0, 0) was used as the axis of the plot, and a diagonal line was added to the plot. As shown in Figure 6, one of the three attributes is plotted in different areas of the two scenic spots, while the other two attributes are plotted in the same areas.

Since “rurality experience activity” is the most important attribute, as it is plotted in quadrant 1 and quadrant 2 for the two scenic spots, it suggests that improvement of efforts and special attention should be given to Dalishu scenic spots while keeping up the good work for the Qingshan scenic spots. However, the attributes of rural tourism service could be characterized as low priority. According to the discrepancy, improving rural tourism service should attract the attention of managers in Qingshan scenic spots. Finally, for the attribute of price level plotted in quadrant 4, it suggests that business managers should pay more attention to keeping the existing level while minimizing the cost.

**Figure 7:**
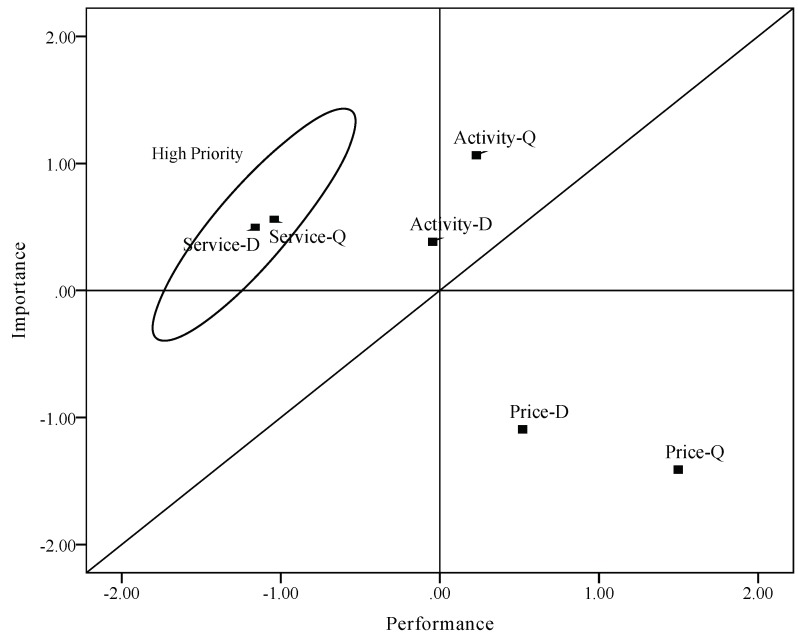
Traditional importance-performance grid (D: Dalishu scenic spots; Q: Qingshan scenic spots).

To compare the revised method with traditional IPA, we computed values of importance and performance rated directly by customers (Table 7). Discrepancy was also acquired by computing standardized value of importance and performance. Then, we plotted these values of importance and performance derived directly on the IPA grid (Figure 7), which used point (0, 0) as the axis of the plot with diagonal line added. Attributes plotted in Figure 7 are distributed in three areas. In this grid, “rurality experience activity” and “rural tourism price level” are shown in the same area with the revised IPA grid. Different from the IPA grid using the revised method, the attribute “rural tourism service” is plotted in the “concentrate here” area, suggesting that these attributes are good candidates for improvement measures. Moreover, according to the results of discrepancy, services in both scenic plots need to be given high priority. This is also a little different from the results from the revised IPA grid.

Considering the importance grid analysis results, “rural tourism service” is categorized as a basic attribute. It means that this attribute has an impact on the overall satisfaction when it is unmet, although it will not enhance its satisfaction when the needs are exceeded. For this reason, Figure 7 seems to have similar plot of attributes with the revised IPA grid. However, according to results of conjoint analysis and the importance grid, the importance of service in these two tourism sites is less than the activity. As a basic attribute, it is more accurately plotted in the third area of the IPA grid.

## 5. Conclusions

This paper presents a new method for measuring the importance and performance of rural tourism products attributes. Instead of using ‘self-stated’ method to measure them directly, this study applies the conjoint analysis method for analyzing the relative importance of attributes. This revised IPA model is employed to identify the category of attributes of rural scenic spots to find appropriate satisfaction enhancing strategies. The study results revealed two attributes on which improvement efforts should be made: “rurality experience activity” in Dalishu and “rural tourism service” in Qingshan scenic spots. Beyond our initial thought, Dalishu scenic spots, which are famous for agriculture experience activities, have a greater discrepant on activity attribute. Combined with the results from importance grid analysis, the importance and utility of “rurality experience activity” need to be emphasized by managers. With respect to Qingshan scenic spots, “rural tourism service” needs to be more focused on by managers, although it is not the high-performance factor. It can be seen that, for the same type of leisure destinations, the improvement focus also appears to be different through this revised IPA approach.

Compared to the traditional IPA technique, the proposed IPA model better shows the importance of attributes based on the conjoint analysis. Moreover, different from other importance measurement methods that focus only on the importance for purchase decision or satisfaction, this study examines the attribute importance twofold: importance in product choice, and importance in delivering satisfaction. Therefore, these results enable managers to evaluate the improvement of attributes more accurately. Furthermore, this study supports previous studies in which a nonlinear relationship exists among satisfaction and attributes. Using the importance grid method, the category of attributes can be acquired, and the relationship among attributes and satisfaction can be obtained. Regarding the survey that was conducted in rural settings, the better sustainable practices are necessary for the development of rural destinations. This study can provide managers of rural tourism destinations with a useful guide on how to enhance overall satisfaction through identifying the factors that have a direct impact on satisfaction, thereby fostering destinations’ profitability and sustainable tourism.

With regard to its limitations, this study can be improved in three aspects. First, only three attributes are used in this revised IPA technique. More attributes should be taken into account in the process of product selection. At the same time, managers also need to consider various factors when seeking improvement. Future research therefore should select more attributes to analyze in order to provide more practical suggestions. Regarding the conjoint analysis method, fractional factorial designs or partial profile design will be suggested for use with a large number of attributes. Second, samples of two rural scenic spots were selected in the present study. The number was not enough for representing the general rural tourism. A sample with a wider range of respondents and more accessible investigation methods are also needed in future studies. Finally, as an effective tool, IPA has been used in tourism research for many years. However, tourist experience is a reflection of tourism products, and the revised IPA model needs to consider the characteristics of tourism products and be examined in more fields for various products in future research.

## Figures and Tables

**Table 1:** Experimental matrix of Box–Behnken design.

Run	Coded Levels		
	$X_{1}$	$X_{2}$	$X_{3}$
1	−1	−1	0
2	−1	1	0
3	1	−1	0
4	1	1	0
5	−1	0	−1
6	−1	0	1
7	1	0	−1
8	1	0	1
9	0	−1	−1
10	0	−1	1
11	0	1	−1
12	0	1	1

**Table 2:** Rural tourism product attributes and levels.

Selected Attributes	Levels	Coding
Rural tourism price level	Worse than expected	−1
	About what expected	0
	Better than expected	1
Rurality experience activity	Worse than expected	−1
	About what expected	0
	Better than expected	1
Rural tourism service	Worse than expected	−1
	About what expected	0
	Better than expected	1

**Table 3:** Demographic of the sample (*n* = 115).

Demographic Variables	Frequency	%
Gender		
Male	41	35.652
Female	74	64.348
Age		
up to 35	49	42.609
36–44	39	33.913
over 46	27	23.478
Monthly income (US dollar)		
below 450	41	35.652
451–900	51	44.348
above 901	23	20.000
Occupation		
tourism-related occupation	40	34.783
tourism-unrelated occupation	51	44.348
student in tourism-related major	9	7.826
student in tourism-unrelated major	12	10.435
retirement	3	2.609

**Table 4:** Validity and reliability of the model.

	Value	Sig
Dalishu scenic spot		
Pearson’s R	0.945	0.000
Kendall’s tau	0.870	0.000
Kendall’s tau for Holdouts	0.333	0.248
Qingshan scenic spot		
Pearson’s R	0.950	0.000
Kendall’s tau	0.818	0.000
Kendall’s tau for Holdouts	0.333	0.248

**Table 5:** Mean implicit and explicit importance ratings of each attribute.

Attributes	Implicit Importance	Explicit Importance
Dalishu scenic spot		
Rural tourism product price level	0.303	3.787
Rurality experience activity	0.418	4.262
Rural tourism service	0.279	4.295
Qingshan scenic spot		
Rural tourism product price level	0.271	3.685
Rurality experience activity	0.396	4.481
Rural tourism service	0.333	4.315

**Table 6:** Importance and performance of rural tourism products attributes.

Attributes	Performance	Importance	Discrepancy
Dalishu scenic spot		
Rural tourism product price level	3.295 (0.520)	0.303 (−0.494)	(1.014)
Rurality experience activity	3.262 (−0.046)	0.418 (1.379)	(−1.425)
Rural tourism service	3.197 (−1.161)	0.279 (−0.885)	(−0.276)
Qingshan scenic spot		
Rural tourism product price level	3.352 (1.498)	0.271 (−1.015)	(2.513)
Rurality experience activity	3.278 (0.229)	0.396 (1.021)	(−0.792)
Rural tourism service	3.204 (−1.041)	0.333 (−0.005)	(−1.035)
Average	3.265	0.388	

**Table 7:** Directly derived importance and performance of rural tourism products’ attributes.

Attributes	Performance	Importance	Discrepancy
Dalishu scenic spot		
Rural tourism product price level	3.295 (0.520)	3.787 (−1.090)	(1.610)
Rurality experience activity	3.262 (−0.046)	4.262 (0.387)	(−0.433)
Rural tourism service	3.197 (−1.161)	4.295 (0.490)	(−1.650)
Qingshan scenic spot		
Rural tourism product price level	3.352 (1.498)	3.685 (−1.407)	(2.905)
Rurality experience activity	3.278 (0.229)	4.481 (1.068)	(−0.839)
Rural tourism service	3.204 (−1.041)	4.315 (0.552)	(−1.592)
Average	3.265	4.138

## References

[1] Kastenholz E., Carneiro M.J., Marques C.P., Lima J. (2012). Understanding and managing the rural tourism experience—The case of a historical village in Portugal. Tour. Manag. Perspect..

[2] Matzler K., Sauerwein E. (2002). The factor structure of customer satisfaction: An empirical test of the importance grid and the penalty-reward-contrast analysis. Int. J. Serv. Ind. Manag..

[3] Poon W.C., Lock-Teng Low K. (2005). Are travellers satisfied with Malaysian hotels?. Int. J. Contemp. Hosp. Manag..

[4] Lancaster K.J. (1966). A new approach to consumer theory. J. Polit. Econ..

[5] Jaccard J., Brinberg D., Ackerman L.J. (1986). Assessing attribute importance: A comparison of six methods. J. Consum. Res..

[6] Oliver R.L. (2010). Satisfaction: A Behavioral Perspective on the Consume.

[7] Martilla J.A., James J.C. (1977). Importance–Performance Analysis. J. Mark..

[8] Ennew C.T., Reed G.V., Binks M.R. (1993). Importance-performance analysis and the measurement of service quality. Eur. J. Mark..

[9] Ford J.B., Joseph M., Joseph B. (1999). Importance-performance analysis as a strategic tool for service marketers: The case of service quality perceptions of business students in New Zealand and the USA. J. Serv. Mark..

[10] Baloglu S., Mangaloglu M. (2001). Tourism destination images of Turkey, Egypt, Greece, and Italy as perceived by US-based tour operators and travel agents. Tour. Manag..

[11] Wade D.J., Eagles P.F. (2003). The use of importance–performance analysis and market segmentation for tourism management in parks and protected areas: An application to Tanzania’s national parks. J. Ecotour..

[12] Croes R., Kubickova M. (2013). From potential to ability to compete: Towards a performance-based tourism competitiveness index. J. Destin. Mark. Manag..

[13] Haemoon O. (2001). Revisiting importance-performance analysis. Tour. Manag..

[14] Louviere J.J., Hout M. (1988). Analyzing Decision Making: Metric Conjoint Analysis.

[15] Myers J.H., Alpert M.I. (1968). Determinant buying attitudes: Meaning and measurement. J. Mark..

[16] Koh S.N., Yoo J.E.J., Boger C.A.J. (2010). Importance-performance analysis with benefit segmentation of spa goers. Int. J. Contemp. Hosp. Manag..

[17] Sethna B.N. (1982). Extensions and Testing of Importance–Performance Analysis. Bus. Econ..

[18] Lee H.S. (2015). Measurement of visitors’ satisfaction with public zoos in Korea using importance-performance analysis. Tour. Manag..

[19] Kuo N.T., Chang K.C., Lai C.H. (2011). Identifying critical service quality attributes for higher education in hospitality and tourism: Applications of the Kano model and importance-performance analysis (IPA). Afr. J. Bus. Manag..

[20] Bacon D.R. (2003). A comparison of approaches to importance-performance analysis. Int. J. Mark. Res..

[21] Azzopardi E., Nash R. (2013). A critical evaluation of importance–performance analysis. Tour. Manag..

[22] Sever I. (2015). Importance-performance analysis: A valid management tool?. Tour. Manag..

[23] Deng W. (2007). Using a revised importance–performance analysis approach: The case of Taiwanese hot springs tourism. Tour. Manag..

[24] Abalo J., Varela J., Manzano V. (2007). Importance values for Importance–Performance Analysis: A formula for spreading out values derived from preference rankings. J. Bus. Res..

[25] Chen K.Y. (2014). Improving importance-performance analysis: The role of the zone of tolerance and competitor performance. The case of Taiwan’s hot spring hotels. Tour. Manag..

[26] Pak R.J. (2016). Combination of importance-performance analysis and response surface methodology for enhancing satisfaction. Int. J. Qual. Reliab. Manag..

[27] Matzler K., Bailom F., Hinterhuber H.H., Renzl B., Pichler J. (2004). The asymmetric relationship between attribute-level performance and overall customer satisfaction: A reconsideration of the importance–performance analysis. Ind. Mark. Manag..

[28] Matzler K., Fuchs M., Schubert A. (2004). Employee satisfaction: Does Kano’s model apply?. Total Qual. Manag. Bus. Excell..

[29] Deng W.J., Chen W.C., Pei W. (2008). Back-propagation neural network based importance–performance analysis for determining critical service attributes. Expert Syst. Appl..

[30] Slevitch L., Oh H. (2010). Asymmetric relationship between attribute performance and customer satisfaction: A new perspective. Int. J. Hosp. Manag..

[31] Mittal V., Ross W.T., Baldasare P.M. (1998). The asymmetric impact of negative and positive attribute-level performance on overall satisfaction and repurchase intentions. J. Mark..

[32] Deng W.J., Kuo Y.F., Chen W.C. (2008). Revised importance–performance analysis: Three-factor theory and benchmarking. Serv. Ind. J..

[33] Neslin S.A. (1981). Linking product features to perceptions: Self-stated versus statistically revealed importance weights. J. Mark. Res..

[34] Green P.E., Rao V.R. (1971). Conjoint measurement for quantifying judgmental data. J. Mark. Res..

[35] Rao V.R. (2014). Applied Conjoint Analysis.

[36] Tamimi N., Sebastianelli R. (2015). The relative importance of e-tailer website attributes on the likelihood of online purchase. Internet Res..

[37] Yen K.H., Chee H.K.L. Empirical examination of AHP and conjoint analysis on casino attributes in Macau. Proceedings of the an International Conference on Public Welfare and Gaming Industry.

[38] Danaher P.J. (1997). Using conjoint analysis to determine the relative importance of service attributes measured in customer satisfaction surveys. J. Retail..

[39] Matzler K., Hinterhuber H.H., Bailom F., Sauerwein E. (1996). How to delight your customers. J. Prod. Brand Manag..

[40] Noriaki K. Life cycle and creation of attractive quality. Proceedings of the 4th QMOD Conference.

[41] Vavra T.G. (1997). Improving Your Measurement of Customer Satisfaction: A Guide to Creating, Conducting, Analyzing, and Reporting Customer Satisfaction Measurement Programs.

[42] Mikulić J., Prebežac D. (2011). Rethinking the importance grid as a research tool for quality managers. Total Qual. Manag. Bus. Excell..

[43] Mikulić J., Prebežac D. (2011). A critical review of techniques for classifying quality attributes in the Kano model. Manag. Serv. Qual. Int. J..

[44] Jin Y., Park Y., Yu J. (2019). An Assessment Model for Evaluating Asymmetric Effects of Attribute-Level Performance on Satisfaction. Sustainability.

[45] Matzler K., Sauerwein E., Heischmidt K. (2003). Importance-performance analysis revisited: The role of the factor structure of customer satisfaction. Serv. Ind. J..

[46] Lai I.K.W., Hitchcock M. (2015). Importance–performance analysis in tourism: A framework for researchers. Tour. Manag..

[47] Zheng L., Liu H. (2013). Evaluating multifunctional agriculture in Dalishu, China: A combined application of SWOT analysis and the analytic network process method. Outlook Agric..

[48] Zheng L., Liu H. (2014). Increased farmer income evidenced by a new multifunctional actor network in China. Agron. Sustain. Dev..

[49] Reichel A., Lowengart O., Milman A. (2000). Rural tourism in Israel: Service quality and orientation. Tour. Manag..

[50] Park D.B., Yoon Y.S. (2009). Segmentation by motivation in rural tourism: A Korean case study. Tour. Manag..

[51] Devesa M., Laguna M., Palacios A. (2010). The role of motivation in visitor satisfaction: Empirical evidence in rural tourism. Tour. Manag..

[52] Kusumawati A.R. Understanding Student Choice Criteria for Selecting an Indonesian Public University: A Conjoint Analysis Approach. Proceedings of the SBS HDR Student Conference.

